# Efficient resuscitation of early-stage viable but non-culturable cells of *Vibrio cholerae* using treatment with proteolytic enzymes

**DOI:** 10.1128/aem.01513-25

**Published:** 2025-12-31

**Authors:** Shin-ichi Miyoshi, Mona Ogasawara, Shiho Niwaki, Rena Sugihara, Basilua Andre Muzembo, Daisuke Imamura

**Affiliations:** 1 Graduate School of Medicine, Dentistry and Pharmaceutical Sciences, Okayama University 12997, Okayama, Japan; 2 Research Center for Intestinal Health Science, Okayama University 12997, Okayama, Japan; 3 Research Institute of Nursing Care for People and Community, University of Hyogo 157679, Akashi, Hyogo, Japan; Anses, Maisons-Alfort Laboratory for Food Safety, Maisons-Alfort, France

**Keywords:** *Vibrio cholerae*, viable but non-culturable, VBNC, protease, proteolytic enzyme

## Abstract

*Vibrio cholerae*, the etiological agent of cholera, is ubiquitous in environmental brackish waters. Exposure to low water temperatures induces the bacterium to enter a viable but non-culturable (VBNC) state. In this study, a stepwise decrease in water temperature to 4°C was found to delay the transition to the non-culturable state compared to an abrupt temperature drop, suggesting that *V. cholerae* cells partially adapt to low temperatures. *V. cholerae* VBNC cells maintained at 4°C gradually lost their ability to revert to a culturable state. However, VBNC cells in the early stage of dormancy were efficiently resuscitated following treatment with proteolytic enzymes, including proteinase K. The abundance of culturable *V. cholerae* cells in brackish estuarine waters was quantified using the most probable number (MPN)–quantitative polymerase chain reaction (qPCR) method. Although culturable cells were undetectable in samples treated with bovine serum albumin, they were estimated at 93 and 1,500 MPN/mL in two water samples collected on different days and pre-incubated with proteinase K. Similarly, the abundance of *Vibrio* species increased markedly following treatment with this enzyme. Additionally, cells of *Vibrio* species were enumerated by the plating method using CHROMagar Vibrio plates. Consistent with the results of the MPN–qPCR method, treatment with proteinase K resulted in over a 100-fold increase in colony formation. Collectively, these findings suggest that treatment with proteinase K is effective for resuscitating and quantifying *V. cholerae* VBNC cells in environmental water samples.

**IMPORTANCE:**

*V. cholerae* enters into a viable but non-culturable (VBNC) state when exposed to low water temperatures. Contamination of food and drinking water with VBNC cells poses a critical public health risk, as these cells retain their virulence but cannot be detected by conventional methods. In this study, we demonstrated that VBNC cells of *V. cholerae* could be efficiently resuscitated by treatment with proteolytic enzymes such as proteinase K, enabling their detection through standard culture-based assays. Environmental brackish water samples were analyzed for *V. cholerae* density using the most probable number (MPN)–quantitative polymerase chain reaction (qPCR) method. While *V. cholerae* was not detected in untreated samples, proteinase K treatment revealed densities of 93 or 1,500 MPN/mL. Therefore, the combination of proteinase K treatment with the MPN–qPCR method offers a promising approach for detecting VBNC bacterial contamination in food, drinking water, and environmental water.

## INTRODUCTION

Bacteria in the genus *Vibrio* (vibrios) are gram-negative, facultative anaerobic rods that are ubiquitous in diverse aquatic environments. However, several species within this genus are notable human pathogens capable of causing both intestinal and extraintestinal infections (1, 2). For instance, *Vibrio cholerae* causes severe watery diarrhea when ingested through contaminated fish, shellfish, or drinking water (1, 2). This species is classified into more than 200 serogroups based on differences in the somatic O-antigen. Among these, only two serogroups (O1 and O139) are responsible for epidemic cholera because of their ability to colonize the surface of the small intestine via toxin-coregulated pili and to produce cholerae toxin, which induces excessive secretion of Cl^−^, followed by H_2_O, Na^+^, K^+^, and HCO_3_^−^, into the intestinal lumen. In contrast, the serogroups non-O1 and non-O139 are also pathogenic to humans but typically cause sporadic, non-epidemic diarrheal disease (1, 2).

In temperate regions, including Japan, the growth and proliferation of *Vibrio* species are strongly influenced by water temperature (3, 4). Typically, vibrios are usually not detected in water samples collected during winter, when water temperatures fall below 10°C, whereas high cell densities are commonly isolated from summer samples, when water temperatures reach approximately 20°C or higher (2–4).

When exposed to environmental stresses such as low water temperatures or nutrient deprivation, gram-negative foodborne human pathogens, including *V. cholerae*, *Vibrio parahaemolyticus*, *Vibrio vulnificus*, *Escherichia coli*, *Salmonella* species, *Shigella* species, *Campylobacter jejuni*, and gram-positive foodborne human pathogens, such as *Bacillus cereus*, *Clostridium perfringens*, and *Listeria monocytogenes*, can enter a viable but non-culturable (VBNC) state (5–7). VBNC cells are metabolically active, exhibit relatively high ATP levels, sustain high membrane potential, and cause stable production of molecular chaperones (5–7). Thus, they are considered alive and capable of expressing various genes, including those associated with toxin production and virulence (5, 6). However, due to cellular damage through exposure to harsh environmental conditions and/or suppression of growth-related genes, these cells are unable to proliferate on conventional culture media (5–7). VBNC cells may be resuscitated and regain their ability to proliferate when exposed to less stressful environment conditions or resuscitation-promoting factors (6, 7). Thus, such cells are thought to be potentially pathogenic and capable of causing infectious diseases upon resuscitation in human hosts. Therefore, quantifying pathogenic bacteria, including those in the VBNC state, in contaminated food and drinking water is crucial for effective infection control and public health protection.

The molecular mechanisms by which bacterial cells enter a VBNC state and subsequently revert to a culturable form remain poorly understood. However, environmental stressors such as low temperature, nutrient starvation, and oxidative stress have been documented to induce the VBNC state (5–7). Conversely, temperature upshift, sodium pyruvate, and amino acids, including glutamate, are known to stimulate the resuscitation of VBNC cells (5–7). Recently, Debnath and Miyoshi (8) demonstrated that the proteolytic enzyme proteinase K can significantly accelerate the recovery of *V. cholerae* VBNC cells to a culturable state. Earlier, Colwell et al. (9) reported that *V. cholerae* VBNC cells could regain culturability within the human intestine. These findings suggest that *V. cholerae* cells entering the human intestine in a VBNC state may be resuscitated to a culturable state upon exposure to gut proteolytic enzymes.

In this study, to elucidate the role of proteolytic enzymes in the resuscitation of *V. cholerae* VBNC cells, we evaluated whether some proteolytic enzymes could promote recovery from the VBNC state and examined also whether a competitive inhibitor of the enzyme could suppress this ability. Furthermore, we investigated whether treatment with proteolytic enzymes could promote the recovery of *V. cholerae* cells from a VBNC state in brackish water samples collected from an estuary.

## MATERIALS AND METHODS

### Bacterial strains and cultivation

We used three *V. cholerae* non-O1/non-O139 strains (BPS64, AN67, and APS1) in this study (4). Strain BPS64 was isolated from freshwater collected at Abe Pond (34°35′30.2″ N, 133°56′35.0″ E) on 9 September 2014, whereas strains AN67 and APS1 were obtained from brackish water sampled at the estuary of Asahi River (34°35′50.6″ N, 133°57′24.9″ E) in Okayama City. Strain AN67 was isolated on 14 September 2015, and strain APS1 was isolated on 20 May 2013.

*V. cholerae* was cultivated in 5 mL of alkaline peptone water (APW; 1% Bacto Peptone [Thermo Fisher Scientific, Waltham, MA, USA], 1% NaCl, pH 8.8). Briefly, cells were pre-cultured at 37°C for 8 h in 5 mL of APW, after which 0.1 mL of the pre-culture was inoculated into 5 mL of fresh APW and cultured at 37°C for 16 h with shaking. The cell density was estimated to be approximately 1 × 10^8^ CFU/mL (range: 0.9–3.1 × 10^8^ CFU/mL) based on plating on CHROMOagar Vibrio plates (CHROMagar, Paris, France).

### Entering the non-culturable state

*V. cholerae* cells from 5 mL cultures were collected by centrifugation at 6,000 × *g* for 10 min at 20°C, washed twice with 5 mL of artificial sea water (ASW; 423 mM NaCl, 9.00 mM KCl, 10.5 mM CaCl_2_, 22.9 mM MgCl_2_, 25.2 mM MgSO_4_, and 2.14 mM NaHCO_3_) (10), and resuspended in 30 mL of ASW. The cell density was then adjusted to 10^7^, 10^5^, or 10^3^ CFU/mL using ASW. The prepared cell suspensions were incubated under static conditions under two different temperature regimes: one maintained at a constant low temperature of 4°C and the other was subjected to a stepwise temperature decrease at weekly intervals, beginning at 20°C and gradually lowered to 15°C, 10°C, and finally 4°C, where it was maintained for the designated duration. In each cell density, two sets of *V. cholerae* cell suspension were prepared.

The number of culturable cells in each suspension was periodically determined using CHROMagar Vibrio plates (4). On designated sampling days, a 0.1 mL aliquot of each cell suspension was withdrawn, serially diluted in phosphate-buffered saline (PBS; 137 mM NaCl, 8.10 mM Na_2_HPO_4_, 2.68 mM KCl, 1.47 mM KH_2_PO; pH 7.4), and inoculated onto two CHROMagar Vibrio plates. The plates were incubated at 37°C for 24 h, after which the resulting colonies were counted. The day on which no colonies appeared was defined as the point at which all bacterial cells had entered a non-culturable state.

### Resuscitation of VBNC cells

After the cells of *V. cholerae* strain BPS64 or AN67 in the suspensions (10^5^ cells/mL) entered a non-culturable state, the cell suspensions were maintained at 4°C for several additional weeks. The resuscitation of VBNC cells in the suspensions was periodically assessed following incubation at 37°C for 24 h, with or without the addition of a proteolytic enzyme. Three proteolytic enzymes exhibiting the highest activities at pH 7.5–8.0 were selected for the experiments: proteinase K (fungal serine protease from *Engyodontium album*; Nacalai Tesque, Kyoto, Japan), trypsin (mammalian serine protease from hog pancreas, Nacalai Tesque), and thermolysin (bacterial metalloprotease from *Bacillus thermoproteolyticus*, Nacalai Tesque). Briefly, each enzyme (0 or 100 µg) was incubated with 1 mL of non-culturable cell suspension of strain BPS64 or AN67 at 37°C for 24 h. Subsequently, a 0.1 mL aliquot was withdrawn, serially diluted in PBS, and plated onto two CHROMagar Vibrio plates. The plates were incubated at 37°C for 24 h, after which colony-forming units were counted.

To examine the inhibitory effect of leupeptin (FUJIFILM Wako Pure Chemical, Osaka, Japan), which is a competitive peptide inhibitor of trypsin and trypsin-like enzymes in both mammals and bacteria (11, 12), trypsin (10 or 50 µg) and leupeptin (0 or 100 µg) were added to 1 mL suspensions of non-culturable cells of strains BPS64 or AN67. The bacterial cell suspensions were incubated at 37°C for 24 h. After incubation, the number of culturable cells resuscitated from the VBNC state was determined by colony enumeration on CHROMagar Vibrio plates.

### Proteolytic activity of trypsin

Trypsin (4 or 20 µg) and leupeptin (0 or 40 µg) were mixed in 0.4 mL of 50 mM Tris-HCl buffer (TB; pH 8.0). Each enzyme mixture was then combined with 0.2 mL of 0.5% azocasein (Merk KGaB, Darmstadt, Germany) prepared in TB. The reaction mixtures were incubated at 37°C for 30 min, after which the proteolytic reactions were terminated by adding 1.4 mL of 5% trichloroacetic acid. The amounts of azo dye released from azocasein through proteolytic digestion were subsequently determined, and the protease units were calculated as previously described (13).

### Estimation of the most probable number of culturable cells

*V. cholerae* strain APS1 was pre-cultivated in APW at 37°C for 16 h with shaking; this was followed by the serial dilution of the pre-culture in APW. An aliquot (0.1 mL) of each diluted pre-culture was inoculated into three test tubes containing 1 mL of APW and incubated at 25°C for 24 h with shaking. After incubation, each test tube showing visible bacterial growth was heated in a boiling water bath for 10 min, rapidly cooled in an ice water bath for 5 min, and then centrifuged at 15,000 × *g* for 5 min at 4°C. The resulting supernatant containing genomic DNA was collected and subjected to quantitative polymerase chain reaction (qPCR) targeting *ompW* (14), which encodes the outer membrane protein (OMP) OmpW, to detect culturable cells of *V. cholerae* strain APS1.

The primer pair ompW-Fq (5′-TTGAAACAACGGCAACCTAC-3′, positions 554–573) and ompW-Rq (5′-ACTTATAACCACCCGCGATC-3′, positions 629–648) was designed to amplify *ompW*, a gene highly specific to *V. cholerae* (14). For qPCR, 1 µL of DNA extract was mixed with 10 µL of Luna Universal qPCR Master Mix (New England Biolabs, Ipswich, MA, USA), primers (20 pmol in 2 µL), and nuclease-free water (7 µL). The reactions (20 µL) were performed using a Thermal Cycler Dice Real Time System Lite (Takara Bio, Kusatsu, Shiga, Japan) with the following conditions: initial denaturation at 98°C for 2 min, followed by 40 cycles of denaturation at 98°C for 5 s, and annealing/extension at 57°C for 20 s. The most probable number (MPN) of culturable cells of strain APS1 was subsequently estimated based on the number of test tubes yielding positive qPCR results for this strain.

### Estimation of culturable *V. cholerae* cells in environmental brackish water using MPN–qPCR

Brackish water samples were collected from the estuary of Asahi River (34°35′50.6″ N, 133°57′24.9″ E) (4) on three different days in 2022: 7 September (water temperature 27.5°C, salinity 1.08%), 26 September (water temperature 24.7°C, salinity 1.19%), and 19 October (water temperature 19.0°C, salinity 1.96%). Sampling was performed at a depth of approximately 30 cm below the water surface using a sterile 250 mL Heyroht water sampler.

Each sample was supplemented with PBS, 100 µg/mL of proteinase K, or bovine serum albumin (BSA, Nacalai Tesque), and incubated at 37°C for 24 h. Following treatment, the samples were serially diluted in APW, and an aliquot (0.1 mL) of each diluted sample was inoculated into three test tubes containing 1 mL of APW and cultured at 37°C for 24 h with shaking. Genomic DNA was subsequently extracted using heat treatment in a boiling water bath, and the *ompW* gene of *V. cholerae* was amplified by qPCR. Then, the MPN of culturable *V. cholerae* cells was estimated. Additionally, the total cell number of culturable vibrios, including *V. cholerae*, was estimated by qPCR with a PCR primer set targeting the 16S rRNA gene; namely, 16S rRNA-Fq (5′-GGTGAGTAATGCCTGGGAAA-3′, positions 109–128) corresponding to the region between V1 and V2 and 16S rRNA-Rq (5′-CTCAGACCAGCTAGGGATCG-3′, positions 277–296) corresponding to the region between V2 and C2 were designed for qPCR.

The culturable vibrios were also estimated by the CHROMagar Vibrio plating method with brackish water samples collected from the estuary of Asahi River (4) on two different days, 21 May (water temperature 20.5°C, salinity 1.08%) and 22 July (water temperature 28.7°C, salinity 1.04%) in 2024.

## RESULTS AND DISCUSSION

### Effects of cell density and incubation temperature on the induction of the viable but non-culturable state

Cells of *V. cholerae* strain BPS64, AN67, or ASP1 were suspended in ASW at a density of 10^7^ CFU/mL and statically incubated at 4°C. The number of culturable cells was periodically quantified. In all three strains, the cells entered a non-culturable state by approximately day 60, as indicated by the absence of colonies on agar plates.

**Fig 1:**
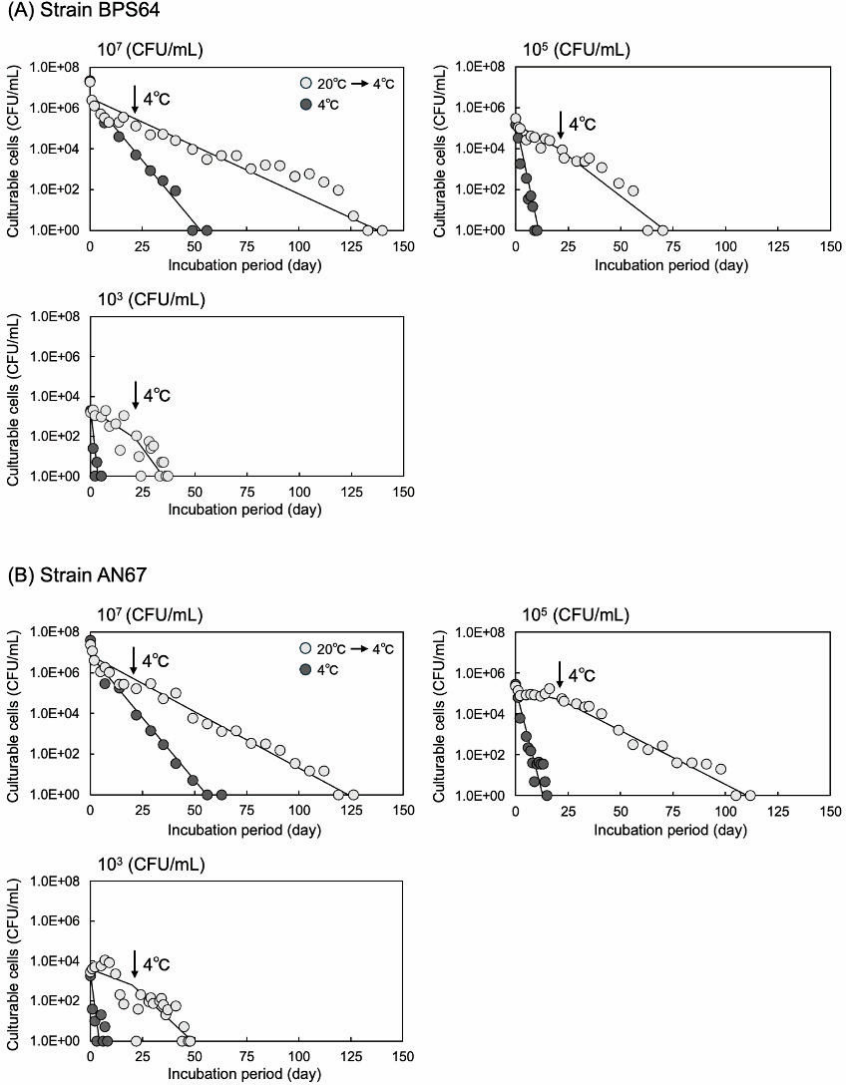
Induction of the VBNC state in *V. cholerae* strain BPS64 and AN67 under cold stress. *V. cholerae* strain BPS64 (**A**) or AN67 (**B**) was suspended in ASW at three initial cell densities (10⁷, 10⁵, and 10³ CFU/mL). The suspensions were incubated statically under two temperature regimes: (i) constant incubation at a low temperature of 4°C and (ii) a stepwise temperature decrease initiated at 20°C and subsequently reduced to 15°C, 10°C, and finally 4°C, where it was maintained for the remainder of the experiment. At designated time points, aliquots were plated on CHROMagar Vibrio medium to quantify culturable cells. The day on which colonies were not observed was defined as the point at which all cells had entered the VBNC state. All experiments were performed in duplicate.

To examine the effect of initial cell density on the transition to a non-culturable state, cells of strains BPS64 and AN67 were suspended in ASW at densities of 10^5^ or 10^3^ CFU/mL and incubated at 4°C. In both cases, a lower initial cell density accelerated the loss of the colony-forming ability. Specifically, strain BPS64 lost culturability by day 10 at 10^5^ CFU/mL and by day 4 at 10^3^ CFU/mL (Fig. 1). The incubation period required for the numbers of culturable cells to decline to one-tenth of the initial counts (*T*_1/10_) was determined. At a cell density of 10^7^ CFU/mL, the *T*_1/10_ value was 8.6 days, whereas it shortened to 2.0 and 1.3 days at 10^5^ and 10^3^ CFU/mL, respectively. Similarly, strain AN67 lost culturability by day 13 at 10^5^ CFU/mL (*T*_1/10_ = 2.6 days) and by day 5 at 10^3^ CFU/mL (*T*_1/10_ = 1.7 days) (Fig. 1). These findings suggest that *V. cholerae* can maintain culturability for a longer period at higher cell densities.

Given the gradual decrease in environmental water temperature, we next examined how temperature changes influenced the induction of cells into the non-culturable state. Cell suspensions of strains BPS64 and AN67 (10^7^, 10^5^, or 10^3^ CFU/mL) were sequentially incubated at 20°C, 15°C, and 10°C, each maintained for 1 week, followed by incubation at 4°C for the designated duration.

As shown in Fig. 1, regardless of the initial bacterial cell density, the time required for the cells to become non-culturable was delayed in both strains under specific conditions. At an initial density of 10^3^ CFU/mL, the number of culturable cells declined more rapidly when incubated at 4°C. However, the transition to the non-culturable state proceeded more slowly when the bacterial cells were subjected to a stepwise temperature decrease than when they were maintained constantly at 4°C. These observations suggest that gradual temperature reduction allowed *V. cholerae* cells to partially adapt to the stress imposed by low water temperature. Nonetheless, this adaptation was ultimately insufficient to prevent entry into the non-culturable state. The molecular mechanisms underlying this partial adaptation to a stepwise temperature decrease remain unclear. However, Datta and Bhadra (15) reported that when the actively growing cells of *V. cholerae* were shifted from 37°C to lower temperatures, the bacterium could adapt down to 15°C by inducing the synthesis of two major cold-shock proteins (Csps). These Csps may therefore play a role in facilitating adaptation to gradual temperature decreases.

### Resuscitation of VBNC cells using treatment with proteolytic enzymes

After entering the non-culturable state, cell suspensions of strains BPS64 (1.1 × 10^5^ cells/mL) and AN67 (0.9 × 10^5^ cells/mL) were maintained at 4°C, and at designated time points, each cell suspension was treated with a proteolytic enzyme (100 µg/mL) at 37°C for 24 h. Subsequently, the number of bacterial cells recovered from the VBNC state was determined using the plating method. In both strains, a notable recovery of colony-forming ability was observed following treatment with proteinase K, particularly up to the 21st day (Table 1). The findings indicated that VBNC cells were clearly resuscitated through proteinase K treatment, as previously documented by Debnath and Miyoshi (8). Two other proteolytic enzymes, trypsin and thermolysin, also significantly promoted cell recovery to a culturable state (Table 1). In contrast, BSA (100 µg/mL) had no effect on the resuscitation of VBNC cells (Table S1). We recently found that, when a small number of culturable *V. cholerae* cells (10 CFU/mL) were added to a mixture of fresh ASW and proteinase K, the increase in the number of culturable cells was not promoted after incubation at 37°C for 32 h (8). Therefore, the extremely increased number of the culturable cells observed in the present study was considered to be not due to the regrowth of pre-existing culturable cells. Collectively, these results suggest that promotion of reversion from the VBNC state is a general characteristic of proteolytic enzymes.

**TABLE 1:** Resuscitation from the VBNC state following treatment with proteolytic enzymes^*a*^

Strain	VBNC state (day)	Culturable cells resuscitated (CFU/mL)			
		PBS	Proteinase K	Trypsin	Thermolysin
BPS64	1	78	1.7 × 10^5^	13.3 × 10^5^	7.4 × 10^5^
	21	<3.0	3.0 × 10^5^	11.5 × 10^5^	5.0 × 10^5^
	42	<3.0	<3.0	<3.0	1,800
	84	<3.0	<3.0	<3.0	<3.0
AN67	8	40	4.8 × 10^5^	26.3 × 10^5^	7.5 × 10^5^
	15	42	3.5 × 10^5^	21.8 × 10^5^	4.5 × 10^5^
	41	<3.0	<3.0	<3.0	<3.0

^*a*^ Experiments were carried out in duplicate.

The density of culturable cells resuscitated by proteolytic enzymes exceeded the initial bacterial cell density (approximately 1.0 × 10^5^ CFU/mL). This observation suggests that *V. cholerae* cells underwent several rounds of cell division prior to entering the non-culturable state during incubation at low water temperatures (20°C, 15°C, 10°C, and 4°C), or that *V. cholerae* cultures grown in APW initially contained numerous numbers of VBNC cells sensitive to proteolytic enzymes.

However, no bacterial cells regained culturability following proteolytic enzyme treatment on the 41st day (strain AN67) or 84th day (strain BPS64) after entering the VBNC state (Table 1). These findings suggest that *V. cholerae* VBNC cells progressively lose their ability to revert to a culturable state through three distinct stages: (i) reversion to culturability by incubation at 37°C without proteolytic enzyme treatment; (ii) resuscitation requiring proteolytic enzyme treatment; and (iii) complete loss of resuscitation, even with proteolytic enzyme treatment.

To determine the prerequisites for proteolytic activity in promoting resuscitation from the VBNC state, we assessed the inhibitory effect of leupeptin, a competitive trypsin inhibitor. On the 11th day (strain BPS64, 3.1 × 10^5^ cells/mL) or 25th day (strain AN67, 2.3 × 10^5^ cells/mL) after entry to the VBNC state, trypsin alone or a mixture of trypsin and leupeptin was added to VBNC cell suspensions and incubated at 37°C for 24 h. The number of cells that regained culturability following trypsin treatment was then determined by counting the colony-forming units on plates. In both strains, treatment with 50 µg/mL of trypsin restored the culturability of VBNC cells to levels comparable to those achieved with 100 µg/mL of trypsin (Table 1). In contrast, treatment with 10 µg/mL of trypsin resulted in an approximately 10-fold reduction in culturable cells, indicating that the effect of trypsin on recovery of the culturability is dose dependent (Table 2). On the other hand, when a mixture of trypsin and leupeptin was applied to VBNC cells, the number of resuscitated cells decreased by 20%–60% compared with the treatment using trypsin alone (Table 2). This indicates that leupeptin significantly inhibited the resuscitative effect of trypsin on non-culturable cells. Consistently, about 90% of the proteolytic activity of trypsin on azocasein was inhibited by leupeptin addition. Collectively, these results suggest that proteolytic activity is closely linked to the resuscitative effect of trypsin on *V. cholerae* VBNC cells.

**TABLE 2:** Inhibitory effects of leupeptin on trypsin activity

Trypsin (µg/mL)	Leupeptin (µg/mL)	Culturable cells resuscitated (CFU/mL)^*a*^		Proteolytic activity (PU/mL)^*b*^
		Strain BPS64	Strain AN67	
10	0	14.5 × 10^4^	9.2 × 10^4^	2.89 ± 0.14
10	100	2.6 × 10^4^	5.6 × 10^4^	0.14 ± 0.04
50	0	11.7 × 10^5^	16.0 × 10^5^	9.17 ± 0.43
50	100	4.8 × 10^5^	9.6 × 10^5^	0.94 ± 0.10

^*a*^ Experiments were carried out in duplicate.

^*b*^ Experiments were carried out three times.

Similarly, Zhao et al. (16) documented that the proteolytic enzyme YeaZ promoted the resuscitation of VBNC cells of a fish pathogen, *Vibrio harveyi*, to a culturable state. In contrast, mutant forms of YeaZ lacking the proteolytic activity failed to induce resuscitation, showing no effect on VBNC cell recovery (16). The molecular mechanisms underlying proteolytic enzyme-mediated resuscitation of VBNC cells remain unknown. However, given the susceptibility of OMPs to proteolytic degradation (17, 18), it is plausible that the proteolysis of these cell surface proteins serves as a trigger for VBNC cell resuscitation. On the other hand, in gram-positive bacteria such as *Micrococcus* species, the enzyme-degrading peptidoglycan has also been shown to stimulate VBNC cell resuscitation (8, 19). Therefore, the degradation of high-molecular-weight structural components such as OMPs and peptidoglycan on the bacterial cell surface plays a key role in initiating VBNC cell resuscitation. Nevertheless, the molecular mechanisms governing the subsequent intracellular signaling processes remain to be elucidated.

### Estimation of *V. cholerae* culturable cells by the MPN–qPCR method

The combination of the MPN method with qPCR, referred to as the MPN–qPCR method, has been applied for the rapid detection and enumeration of bacteria in various environmental samples, including air, water, and shellfish (20, 21). In this study, we evaluated the applicability of the MPN–qPCR method for estimating the abundance of culturable *V. cholerae* cells in environmental water.

A pre-culture of *V. cholerae* strain APS1 grown in APW was serially diluted and inoculated into three test tubes containing APW, followed by cultivation at 25°C for 24 h. After cultivation, genomic DNA was extracted from the tube showing visible bacterial growth. The extracted DNA was then subjected to qPCR targeting the *ompW* gene, and the MPN of culturable cells was estimated based on the number of test tubes yielding positive results. The density of culturable cells of strain APS1 in the pre-culture sample was estimated at 9.3 × 10^7^ MPN/mL (range: 1.5–38.0 × 10^7^ cells/mL). In contrast, the plating method using CHROMagar Vibrio plates measured the density of culturable cells in the same pre-culture sample as 17.0 × 10^7^ CFU/mL. No significant difference was observed between bacterial cell counts obtained using the plating and MPN–qPCR method, confirming the reliability of the MPN–qPCR method for quantifying culturable *V. cholerae* cells in environmental samples.

Cells of strain APS1 (2.6 × 10^7^ MPN/mL) that had entered the VBNC state were subsequently maintained at 4°C for 4 weeks. At designated time points, the VBNC cells were treated with PBS or proteinase K (100 µg/mL) at 37°C for 24 h. The number of resuscitated cells was then quantified through the MPN–qPCR targeting the *V. cholerae*-specific *ompW* gene (Table 3). On days 6 and 19, proteinase K treatment successfully promoted the resuscitation of VBNC cells, whereas PBS treatment did not. Therefore, the combined application of the MPN–qPCR method and proteinase K treatment (hereafter referred to as the improved MPN–qPCR method) appears to be a useful approach for the rapid estimation of *V. cholerae* VBNC cells.

**TABLE 3:** Resuscitation of VBNC cells following treatment with or without proteinase K

VBNC state (day)	Culturable cells resuscitated (MPN/mL)	
	PBS	Proteinase K
6	9.3	5.6 × 10^7^
19	NT^*a*^	14.0 × 10^7^
27	NT^*a*^	<0.3

^*a*^ Not tested.

A few numbers of culturable cells might be present in the culture of strain APS1, which was judged to have entered the VBNC state, and might multiply during the proteinase K treatment. However, as we recently reported (8), no stimulation of the bacterial growth was observed even when a small number of culturable *V. cholerae* cells (10 CFU/mL) were added to proteinase K (100 µg/mL) and incubated at 37°C for 32 h. Therefore, it may be concluded that the culturable cells detected by the MPN–qPCR method were the cells recovered from the VBNC state.

However, by day 27, no significant recovery from the non-culturable state was observed, even following treatment with proteinase K. This suggests that only early-stage VBNC cells remain to be responsive to proteinase K.

### Application of the improved MPN–qPCR method to estimate *V. cholerae* in environmental brackish water

We applied the improved MPN–qPCR method to quantify *V. cholerae* cells in brackish water samples collected from the estuary of Asahi River in Okayama City (Table 4). In the sample collected on 7 September (water temperature 27.5°C), culturable *V. cholerae* cells were not detected following treatment with PBS (<0.3 MPN/mL). In contrast, the number of culturable cells markedly increased to 1,500 MPN/mL (range: 300–4,400 cells/mL) after treatment with proteinase K. Similarly, in the sample collected on 26 September (water temperature 24.7°C), *V. cholerae* was detected at a density of 93 MPN/mL (range: 15–380 cells/mL) following incubation with proteinase K, whereas no cells were detected after incubation with PBS or BSA (<0.3 MPN/mL). These results suggest that nearly all *V. cholerae* cells in the water samples were in the VBNC state, despite water temperatures exceeding 20°C. In contrast, *V. cholerae* was not detected in the sample collected on 19 October (water temperature 19.0°C), even after proteinase K treatment, suggesting that the VBNC cells may have transitioned to a non-responsive dormant state.

**TABLE 4:** Enumeration of culturable *V. cholerae* and other vibrios species in brackish water^*a*^

Date of water collected	Treatment	Culturable cells estimated	
		*V. cholerae*	Vibrios
MPN–qPCR (MPN/ mL)			
7 September 2022	PBS or BSA	<0.3	1,500
	Proteinase K	1,500	≥24,000
26 September 2022	PBS or BSA	<0.3	150 930
	Proteinase K	93	≥24,000
19 October 2022	PBS or BSA	<0.3	93
	Proteinase K	<0.3	≥24,000
Plating (CFU/mL)			
21 May 2024	PBS or BSA	NT^*a*^	17 47
	Proteinase K	NT^*a*^	1,600
22 July 2024	PBS or BSA	NT^*a*^	160 220
	Proteinase K	NT^*a*^	87,000

^*a*^ Not tested.

We further performed the improved MPN–qPCR targeting the 16S rRNA gene to quantify the total number of vibrios (Table 4). Proteinase K treatment significantly increased the number of *Vibrio* species detected, demonstrating its effectiveness in resuscitating both *V. cholerae* and other *Vibrio* species. To validate the results obtained using the improved MPN–qPCR method, we concurrently employed the CHROMagar Vibrio plating method to estimate vibrios in the brackish water samples. Consistent with the improved MPN–qPCR results, the numbers of colonies formed on the plates increased approximately 340- to 540-fold following proteinase K treatment (Table 4). This finding suggests that over 99% of the vibrio cells in the collected water samples were in the VBNC state. Collectively, these results demonstrate that the improved MPN–qPCR method developed in this study is well suited for the rapid quantification of both VBNC and culturable cells of *V. cholerae* and other *Vibrio* species in environmental water samples.

In conclusion, this study demonstrated that *Vibrio* species, including *V. cholerae*, were present in environmental brackish water in the VBNC state, far exceeding the numbers determined by conventional culture-based methods. In addition, early-stage VBNC cells were efficiently resuscitated into culturable forms upon exposure to proteolytic enzymes such as proteinase K. Given that proteolytic enzymes are common digestive enzymes in the intestines of fish (22), the shellfish hepatopancreas (23), and the human gastrointestinal tract, it is plausible that *V. cholerae* VBNC cells may revert to a culturable state within the bodies of fish, shellfish, and humans. Therefore, quantifying both culturable and VBNC *V. cholerae* is essential for accurate risk assessment of seafood products and environmental water sources. The improved MPN–qPCR method developed in this study is relatively straightforward, as it requires only a proteolytic enzyme pre-treatment in addition to the conventional method. This simplicity suggests that the improved MPN–qPCR method can be effectively applied to detect contamination by *V. cholerae*, including VBNC cells. However, environmental water may contain various active microorganisms that can proliferate or interact during the 37°C for 24 h pre-treatment process. Therefore, before integrating the improved MPN–qPCR method into the environmental monitoring system, optimization of the pre-treatment conditions, particularly temperature and incubation time, is necessary.

## Data Availability

The data that support the findings of this study are available from the corresponding author upon reasonable request.

## SUPPLEMENTAL MATERIAL

Table S1 aem.01513-25-s0001.docx No effect of BSA on resuscitation from the VBNC state.

ASM does not own the copyrights to Supplemental Material that may be linked to, or accessed through, an article. The authors have granted ASM a non-exclusive, world-wide license to publish the Supplemental Material files. Please contact the corresponding author directly for reuse.

## References

[1] Janda JM, Powers C, Bryant RG, Abbott SL. 1988. Current perspectives on the epidemiology and pathogenesis of clinically significant Vibrio spp. Clin Microbiol Rev 1:245–267. doi:10.1128/CMR.1.3.245 3058295 PMC358049

[2] Chakraborty S, Nair GB, Shinoda S. 1997. Pathogenic vibrios in the natural aquatic environment. Rev Environ Health 12:63–80. doi:10.1515/reveh.1997.12.2.63 9273923

[3] Waidner LA, Potdukhe TV. 2023. Tools to enumerate and predict distribution patterns of environmental Vibrio vulnificus and Vibrio parahaemolyticus Microorganisms 11:2502. doi:10.3390/microorganisms11102502 37894160 PMC10609196

[4] Miyoshi S, Kurata M, Hirose R, Yoshikawa M, Liang Y, Yamagishi Y, Mizuno T. 2024. Isolation of Vibrio cholerae and Vibrio vulnificus from estuarine waters, and genotyping of V. vulnificus isolates using loop-mediated isothermal amplification. Microorganisms 12:877. doi:10.3390/microorganisms12050877 38792707 PMC11124270

[5] Oliver JD. 2010. Recent findings on the viable but nonculturable state in pathogenic bacteria. FEMS Microbiol Rev 34:415–425. doi:10.1111/j.1574-6976.2009.00200.x 20059548

[6] Zhao X, Zhong J, Wei C, Lin C-W, Ding T. 2017. Current perspectives on viable but non-culturable state in foodborne pathogens. Front Microbiol 8:580. doi:10.3389/fmicb.2017.00580 28421064 PMC5378802

[7] Zhang X-H, Ahmad W, Zhu X-Y, Chen J, Austin B. 2021. Viable but nonculturable bacteria and their resuscitation: implications for cultivating uncultured marine microorganisms. Mar Life Sci Technol 3:189–203. doi:10.1007/s42995-020-00041-3 37073345 PMC10077291

[8] Debnath A, Miyoshi S-I. 2021. The Impact of protease during recovery from viable but non-culturable (VBNC) state in Vibrio cholerae Microorganisms 9:2618. doi:10.3390/microorganisms9122618 34946219 PMC8707003

[9] Colwell RR, Brayton P, Herrington D, Tall B, Huq A, Levine MM. 1996. Viable but non-culturable Vibrio cholerae O1 revert to a cultivable state in the human intestine. World J Microbiol Biotechnol 12:28–31. doi:10.1007/BF00327795 24415083

[10] Asakura H, Ishiwa A, Arakawa E, Makino S, Okada Y, Yamamoto S, Igimi S. 2007. Gene expression profile of Vibrio cholerae in the cold stress-induced viable but non-culturable state. Environ Microbiol 9:869–879. doi:10.1111/j.1462-2920.2006.01206.x 17359259

[11] Aoyagi T, Takeuchi T, Matsuzaki A, Kawamura K, Kondo S, Hamada M, Maeda K, Umezawa H. 1969. Leupeptins, new protease inhibitors from actinomycetes. J Antibiot 22:283–286. doi:10.7164/antibiotics.22.283 5810993

[12] Saino T, Someno T, Ishii S-I, Aoyagi T, Umezawa H. 1988. Protease-inhibitory activities of leupeptin analogues. J Antibiot 41:220–225. doi:10.7164/antibiotics.41.220 2965694

[13] Miyoshi N, Shimizu C, Miyoshi S, Shinoda S. 1987. Purification and characterization of Vibrio vulnificus protease. Microbiol Immunol 31:13–25. doi:10.1111/j.1348-0421.1987.tb03064.x 3295490

[14] Nandi B, Nandy RK, Mukhopadhyay S, Nair GB, Shimada T, Ghose AC. 2000. Rapid method for species-specific identification of Vibrio cholerae using primers targeted to the gene of outer membrane protein OmpW. J Clin Microbiol 38:4145–4151. doi:10.1128/JCM.38.11.4145-4151.2000 11060082 PMC87555

[15] Datta PP, Bhadra RK. 2003. Cold shock response and major cold shock proteins of Vibrio cholerae. Appl Environ Microbiol 69:6361–6369. doi:10.1128/AEM.69.11.6361-6369.2003 14602587 PMC262262

[16] Zhao R, Chen J, Wang Y, Li Y, Kong X, Han Y. 2020. Proteolytic activity of Vibrio harveyi YeaZ is related with resuscitation on the viable but non-culturable state. Lett Appl Microbiol 71:126–133. doi:10.1111/lam.13304 32349168

[17] Coleman JL, Crowley JT, Toledo AM, Benach JL. 2013. The HtrA protease of Borrelia burgdorferi degrades outer membrane protein BmpD and chemotaxis phosphatase CheX. Mol Microbiol 88:619–633. doi:10.1111/mmi.12213 23565798 PMC3641820

[18] Tsaplina O, Demidyuk I, Artamonova T, Khodorkovsky M, Khaitlina S. 2020. Cleavage of the outer membrane protein OmpX by protealysin regulates Serratia proteamaculans invasion. FEBS Lett 594:3095–3107. doi:10.1002/1873-3468.13897 32748449

[19] Mukamolova GV, Murzin AG, Salina EG, Demina GR, Kell DB, Kaprelyants AS, Young M. 2006. Muralytic activity of Micrococcus luteus Rpf and its relationship to physiological activity in promoting bacterial growth and resuscitation. Mol Microbiol 59:84–98. doi:10.1111/j.1365-2958.2005.04930.x 16359320

[20] Orlofsky E, Benami M, Gross A, Dutt M, Gillor O. 2015. Rapid MPN-Qpcr screening for pathogens in air, soil, water, and agricultural produce. Water Air Soil Pollut 226:303. doi:10.1007/s11270-015-2560-x

[21] Walker DI, Fok BCT, Ford CL. 2020. A qPCR-MPN method for rapid quantification of Escherichia coli in bivalve molluscan shellfish. J Microbiol Methods 178:106067. doi:10.1016/j.mimet.2020.106067 32980334

[22] Kim M, Jeong Y. 2013. Purification and characterization of a trypsin-like protease from Flatfish (Paralichthys olivaceus) intestine. J Food Biochem 37:732–741. doi:10.1111/j.1745-4514.2012.00672.x

[23] Song C, Shi Y, Meng X, Wu D, Zhang L. 2020. Identification of a novel alkaline serine protease from gazami crab (Portunus trituberculatus) hepatopancreas and its hydrolysis of myofibrillar protein. Int J Biol Macromol 155:403–410. doi:10.1016/j.ijbiomac.2020.03.179 32229212

