# Supplementary material for: Efficient resuscitation of early-stage viable but non-culturable cells of Vibrio cholerae using treatment with proteolytic enzymes
Source: Appl Environ Microbiol. 2025 Dec 31;92(1):e01513-25. doi: 10.1128/aem.01513-25 (PMC12838185; doi:10.1128/aem.01513-25)
Supplement: Table S1 — No effect of BSA on resuscitation from the VBNC state. [file aem.01513-25-s0001.docx]

**Supplementary Materials**

| Table S1. No effect of BSA on resuscitation from the VBNC state^a^ | | | | | |
| --- | --- | --- | --- | --- | --- |
| Strain | VBNC state  (day) | Culturable cells resuscitated (CFU/mL) | | | |
|  |  | PBS | BSA | Trypsin | Thermolysin |
| BPS64 | 23 | < 3.0 | < 3.0 | 5.9 x 10^5^ | NT^b^ |
| AN67 | 2 | 0.2 x 10^5^ | 0.1 x 10^5^ | NT^b^ | 8.8 x 10^5^ |

^a^ Experiments were carried out in duplicate.

^b^ Not tested.
